# Identification of a putative competitive endogenous RNA network for lung adenocarcinoma using TCGA datasets

**DOI:** 10.7717/peerj.6809

**Published:** 2019-04-23

**Authors:** Yuanyong Wang, Tong Lu, Yang Wo, Xiao Sun, Shicheng Li, Shuncheng Miao, Yanting Dong, Xiaoliang Leng, Wenjie Jiao

**Affiliations:** Department of Thoracic Surgery, Affiliated Hospital of Qingdao University, Qingdao, Shandong, China

**Keywords:** ceRNA network, Lung adenocarcinoma, Prognosis, microRNA, lncRNA

## Abstract

The mechanisms underlying the oncogenesis and progression of lung adenocarcinoma (LUAD) are currently unclear. The discovery of competitive endogenous RNA (ceRNA) regulatory networks has provided a new direction for the treatment and prognosis of patients with LUAD. However, the mechanism of action of ceRNA in LUAD remains elusive. In the present study, differentially expressed mRNAs, microRNAs (miRs) and long non-coding RNAs from the cancer genome atlas database were screened. CeRNAs for LUAD were then identified using online prediction software. Among the ceRNAs identified, family with sequence similarity 83 member A (FAM83A), miR-34c-5p, KCNQ1OT1 and FLJ26245 were observed to be significantly associated with the overall survival of patients with LUAD. Of note, FAM83A has potential significance in drug resistance, and may present a candidate biomarker for the prognosis and treatment of patients with LUAD.

## Introduction

Lung cancer accounts for the highest morbidity and mortality rates worldwide (Siegel, Miller & Jemal, 2018). Lung cancer can be divided into two pathological forms, consisting of non-small cell lung cancer and small cell lung cancer; the former of which can be divided into lung adenocarcinoma (LUAD), lung squamous cell carcinoma (LSCC) and large cell lung cancer (Qin et al., 2018). Recent epidemiological studies have demonstrated that in many countries, including China, the incidence of LUAD is higher than that of LSCC in both smoking and non-smoking populations, and accounts for ∼50% of all lung cancers (Patel et al., 2017). In some countries, the incidence of LUAD in women has exceeded that of men, and it is the most common pathological subtype of lung cancer in young people (Patel et al., 2017). Due to the lack of research investigating the molecular mechanisms underlying the pathogenesis of LUAD, no effective targeted therapies have thus far been discovered, and the treatment success rate is not optimal. Therefore, there is an urgent need for the identification of accurate indicators for the tumorigenesis and development of LUAD.

Recent studies have identified tumor markers, such as CA-199, CA-125, CYFRA21-1 and carcinoembryonic antigen, which are used to guide LUAD treatment (Ghosh et al., 2013; Jiang, Wang & Xu, 2018; Minami et al., 2017; Shintani et al., 2017; Sone et al., 2017). Despite this, the nosogenesis of LUAD remains unclear. In addition, there is a current lack of highly sensitive and specific markers for the diagnosis of early-stage LUAD. Recently, long non-coding RNAs (lncRNAs) have become novel and popular markers for detecting tumors in the bloodstream, which could allow for the dynamic monitoring of tumor cell invasion, activity and patient prognosis (Tian et al., 2018b; Zhao et al., 2018). In addition, lncRNAs have been found to exert multiple functional roles in lung cancer (Li et al., 2018; Qian et al., 2018; Yu et al., 2018; Zhang et al., 2018b). Therefore, identifying lncRNAs associated with LUAD and investigating their molecular mechanisms and clinical effects may be an important step in understanding the development and progression of LUAD.

The Cancer Genome Atlas (TCGA) database holds a large quantity of high-throughput sequencing information, in addition to non-coding RNAs, from patients with LUAD that is available for scientific researchers and clinical doctors to use. A previously published study has identified abnormally expressed genes in LUAD based on TCGA data (Deng et al., 2018). Significantly altered microRNAs (miRs/miRNAs) were also identified, that were found to exert important functions as oncogenes or tumor suppressors (Gao et al., 2018; Liu et al., 2017). Compared with miRNAs, the discovery of LUAD-associated lncRNAs are still at a preliminary stage, but it has been demonstrated that they serve diverse and complex roles in tumorigenesis (Klingenberg et al., 2018). Competitive endogenous RNA (ceRNA), a relatively new RNA regulation theory, hypothesizes that miRNAs may induce or inhibit the expression of their target genes by binding to the 3′-untranslated region (UTR) of mRNAs and ultimately control their activity (Park et al., 2018; Zhang et al., 2018c). Thus, the same miRNA sequence competes for the 3′-UTR region of different genes at the time of transcription, thereby forming a complex RNA regulatory network that may affect a number of cellular physiological processes (Salmena et al., 2011). As such, lncRNAs may not only regulate the cell phenotype, but also regulate miRNAs to serve as “sponges” that attenuate the direct regulation of mRNA by miRNAs (Tian, Qiu & Qiu, 2018a; Xu et al., 2018). Taking this into account, the present study investigated abnormally expressed lncRNAs and miRNAs in 570 LUAD samples from the TCGA database. Importantly, a ceRNA network of LUAD was generated, which may enable a greater understanding of the functional role of lncRNAs in LUAD.

## Materials and Methods

### Patients and samples from the TCGA database

The Cancer Genome Atlas data is divided into three levels; level 3 data is publicly downloaded and contains gene expression information (Vrba & Futscher, 2018). All available level 3 RNA expression data from 513 LUAD cancer cases and 57 paracancerous cases were obtained from the TCGA database for the purposes of the present study. Data exclusion criteria were as follows: (i) No obvious clinical characteristics (*n* = 135); (ii) duplicate sample data (*n* = 2); and (iii) a total survival time of >2,000 days (*n* = 12). Overall, a total of 374 LUAD samples were collected for the purposes of this study. According to the histopathological results, these data were divided into early stage (IA and IB), middle stage (IIA and IIB) and late stage (IIIA, IIIB and IV), while 56 adjacent normal lung tissue samples served as the control group (one case was excluded due to data duplication). This study did not require ethics approval as the patient data were obtained from the TCGA database.

### RNAsequence data precessing and differential expression analysis

The raw RNA sequencing (mRNA, miRNA and lncRNA) reads were post-processed and normalized using the Fragments per kilobase of per million fragments mapped method.

**Figure 1:**
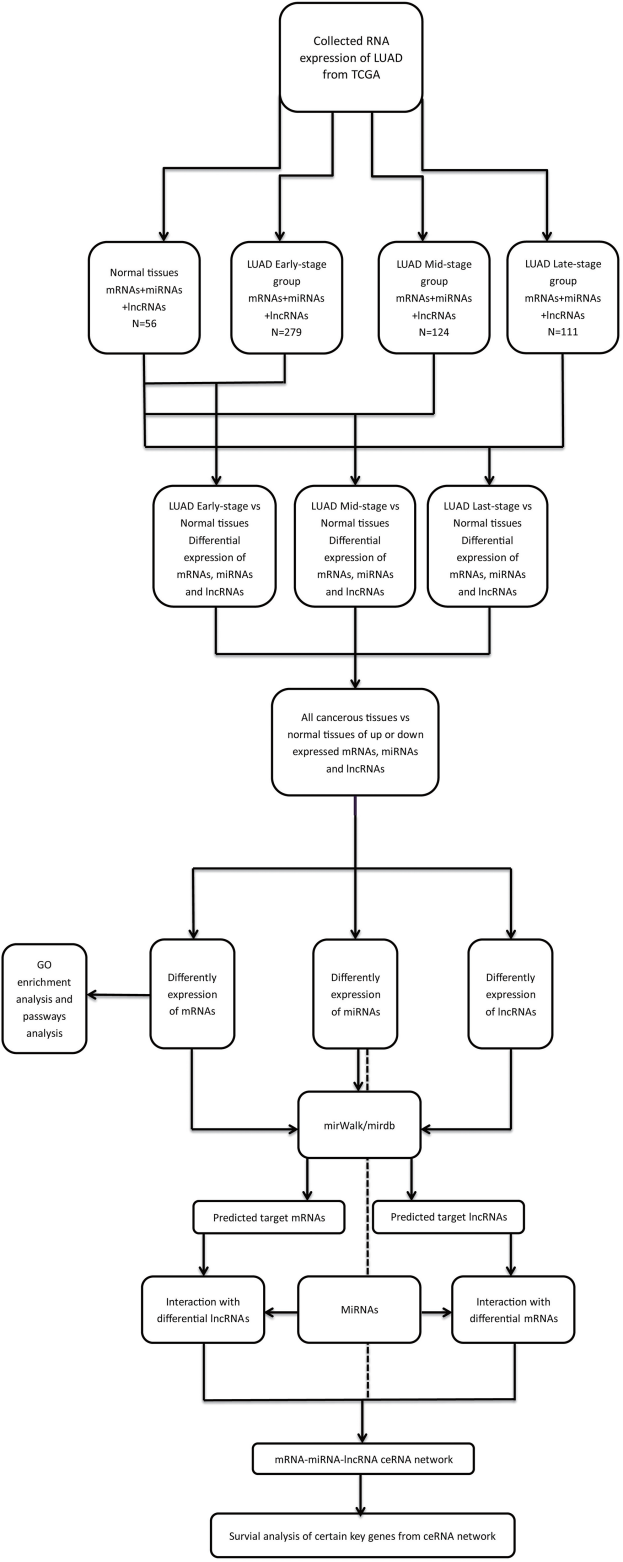
The bioinformatics analysis process.

*Limma* package in R (R Core Team, 2018) was used to identify the differentially expressed mRNAs (DEmRNAs), miRNAs (DEmiRNAs) and lncRNAs (DElncRNAs) between the LUAD and adjacent-normal tissues (Ritchie et al., 2015). A false discovery rate (FDR) < 0.05 and fold change > 2 were considered as thresholds for indicating differential RNA expression. The bioinformatics analysis process was shown in Fig. 1. The heat map and volcano plots were visualized using the *ggplot2* packages in R (Ito & Murphy, 2013).

### Differential gene function and pathway analysis

The Gene Ontology (GO) database (http://www.geneontology.org) was used to provide GO terms for DEmRNAs, and to identify significant biological functions (The Gene Ontology Consortium, 2017). The Kyoto Encyclopedia of Genes and Genomes (KEGG; http://www.kegg.jp/) (Kanehisa et al., 2008) was used to determine the signal transduction pathways and metabolic pathways for up-regulated and down-regulated differentially expressed genes in LUAD (FDR < 0.05).

### Identification and construction of the ceRNA network

Differentially expressed miRNA sequences were identified using mirBase (http://www.mirbase.org/) (Kozomara & Griffiths-Jones, 2014). The predicted mRNA target genes of the miRNAs identified in the present study were confirmed using starBase V3.0 (http://starbase.sysu.edu.cn/) (Li et al., 2014) and miRbase. StarBase was also used to predict miRNAs targeted by lncRNAs. Both miRbase and starBase were used to construct predicted mRNA–miRNA and miRNA–lncRNA associations, respectively. Regarding mRNA activity and expression regulation, it is thought that lncRNAs can adsorb miRNAs via a “sponge effect,” suggesting that miRNAs are competitively regulated by both their corresponding lncRNAs and mRNAs. Therefore, an mRNA–miRNA–lncRNA ceRNA network was constructed in the current study (up-regulated or down-regulated fold change > 2.5; FDR < 0.05) (Liu et al., 2018). HERMES (http://califano.c2b2.columbia.edu/hermes/) also helped us eliminate the vast majority of indirect interactions typically inferred by pairwise analysis and construct ceRNA (Talos et al., 2017). Cytoscape (version 3.6.1) was used to construct an interactive visual ceRNA network.

### Clinical significance of the ceRNA network

Following construction of the predicted ceRNA network, the clinical significance of this network with patient outcomes was subsequently assessed. The correlation among mRNA, miRNAs, lncRNAs and the survival of patients from the TCGA database was determined using a Cox proportional hazards regression model. The effect of mRNAs, miRNAs and lncRNAs on patient survival was statistically analyzed by Cox univariate regression analysis (*P* < 0.05), and a Kaplan–Meier survival curve of LUAD patients was also constructed.

### Cell culture

The normal lung cancer cell line, BEAS-2B, and two LUAD cell lines, A549 and H1299, were purchased from MssBio Co., Ltd. (Guangzhou, China) and the Chinese Academy of Sciences Cell Bank (Shanghai, China). The BEAS-2B was cultured in bronchial epithelial growth medium without fetal bovine serum (FBS). LUAD cancer cell lines were maintained in Dulbecco’s modified Eagle’s medium (Gibco; Thermo Fisher Scientific, Inc., Waltham, MA, USA) with 10% FBS. All cell lines were maintained at 37 °C and 5% CO_2_.

### Total RNA extraction and quantitative-polymerase chain reaction

Total RNA was extracted from lung tissues and cultured cells using TRIzol reagent (Thermo Fisher Scientific, Inc., Waltham, MA, USA). RNA quality was determined using the NanoDrop 2000 spectrophotometer (Thermo Fisher Scientific, Inc., Waltham, MA, USA) and only samples with an A260/A280 ratio of 1.8–2.1 were used. Total RNA was then reverse transcribed to cDNA (Takara Biotechnology Co., Ltd., Dalian, China). quantitative-polymerase chain reaction (qPCR) was performed using an Applied Biosystems 7500 Real-Time PCR system (Applied Biosystems; Thermo Fisher Scientific, Inc., Waltham, MA, USA) and SYBR Green (Takara Biotechnology Co., Ltd., Dalian, China). The primers were as follows: Family with sequence similarity 83 member A (FAM83A), also known as BJ-TSA-9, sense, 5′-CCAGGGCTGACTTTAGTGACAACG-3′, and antisense, 5′-GCCTCCACCGAGGACAAGAAG-3′. The qPCR thermal cycling parameters were as follows: 95 °C for 5 min followed by 40 cycles of 95 °C for 20 s and 63 °C for 30 s. GAPDH was used as an internal control. Fold changes in FAM83A expression between tumor and normal tissues were calculated using the 2^−ΔΔCt^ method for each sample in triplicate (Livak & Schmittgen, 2001).

### Statistical analysis

Statistical analysis was performed using SPSS 23.0 (IBM Corp., Armonk, NY, USA). *P* < 0.05 was considered to indicate a statistically significant difference. FDR < 0.05 was set as a default threshold to control the significance level. Univariate Cox proportional hazards regression was used to identify ceRNAs associated with the overall survival of patients.

## Results

### Differentially expressed genes and their GO and pathway analysis

**Figure 2:**
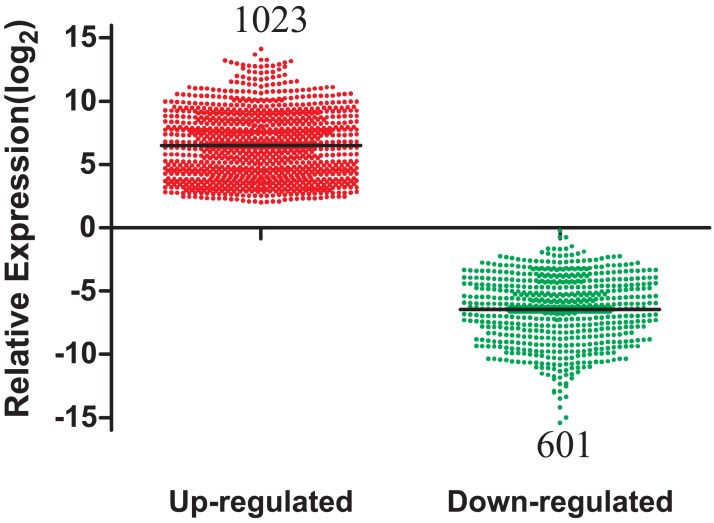
The number of differentially expressed genes collected from the TCGA database among LUAD tumor groups and control groups. TCGA, The Cancer Genome Atlas; LUAD, lung adenocarcinoma.

A total of 1,624 DEmRNAs between LUAD tissues and adjacent normal controls from the TCGA database were identified in Fig. 2. All significantly differentially expressed genes are shown in Supplementary Table Sheet 1 (FDR < 0.05; fold change > 2). In addition, these differentially expressed genes were analyzed using the GO database and were enriched to form a measure of the significance of a function (Fig. S1). In the biological process ontology, signal transduction and cellular processes were the most enriched terms. In cellular components ontology, membrane and extracellular region were the most enriched terms. In molecular function ontology, the most enriched terms were binding and catalytic activities. This provided a definitive functional description of genes differentially expressed in LUAD. Subsequent pathway analysis of these 1,624 differentially expressed genes was performed. The most enriched network corresponding to upregulated transcripts was “cell cycle,” while the most enriched network corresponding to the downregulated transcripts was “neuroactive ligand-receptor interaction.” Additional signaling pathways identified are also shown in Fig. S2.

### Differentially expressed miRNAs and lncRNAs in LUAD and construction of the ceRNA network

**Figure 3:**
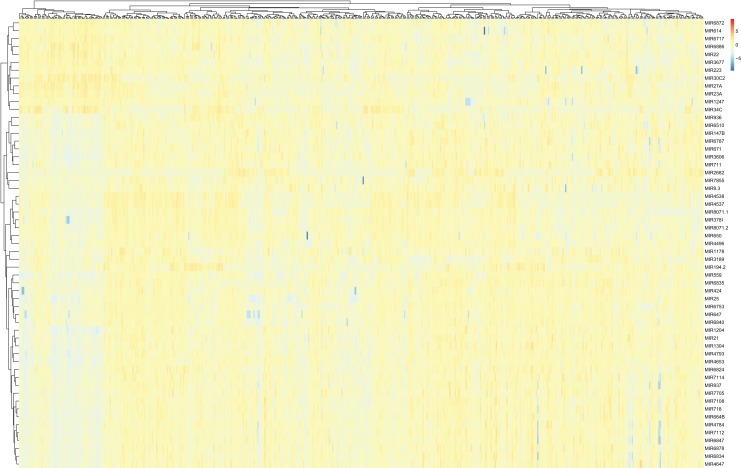
A heat map of differentially expressed miRNAs between LUAD groups and controls from the TCGA database. Red, upregulation; Blue, downregulation. miRNA, microRNA; LUAD, lung adenocarcinoma; TCGA, The Cancer Genome Atlas.

Differentially expressed miRNAs and lncRNAs between LUAD samples and adjacent non-cancerous tissues from the TCGA database were then identified (absolute fold change > 2; *P* < 0.05). A total of 107 miRNAs were found to be differentially expressed between the tumor and control groups, and were selected for the subsequent generation of the ceRNA network (Fig. 3).

**Figure 4:**
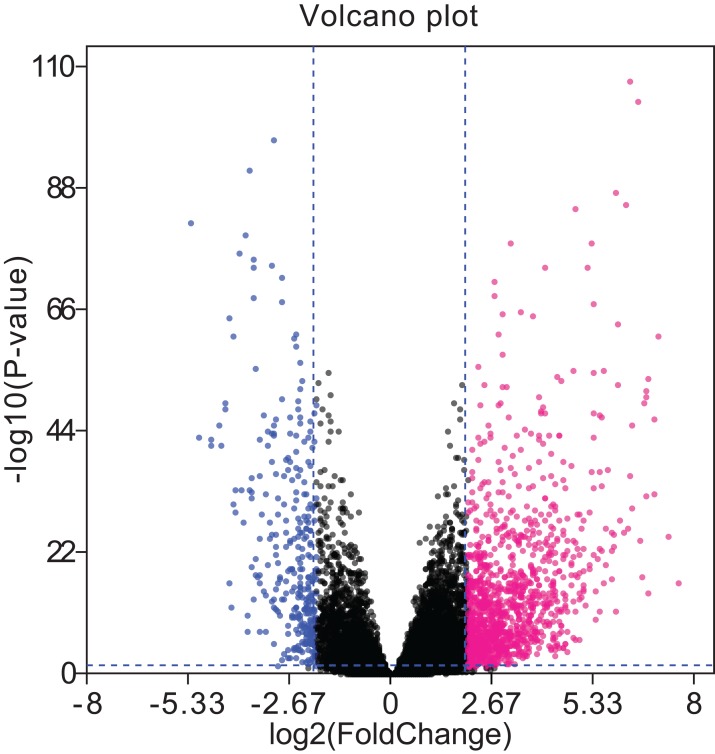
Volcano plots of aberrantly expressed lncRNAs. A total of 426 upregulated lncRNAs showed significantly abnormal expression, while 253 were downregulated (fold change ≥ 2; *P* < 0.05, FDR < 0.05). The red and blue nodes correspond to upregulated and downregulated lncRNAs, respectively. LncRNA, long non-coding RNA; FDR, false discovery rate.

**Figure 5:**
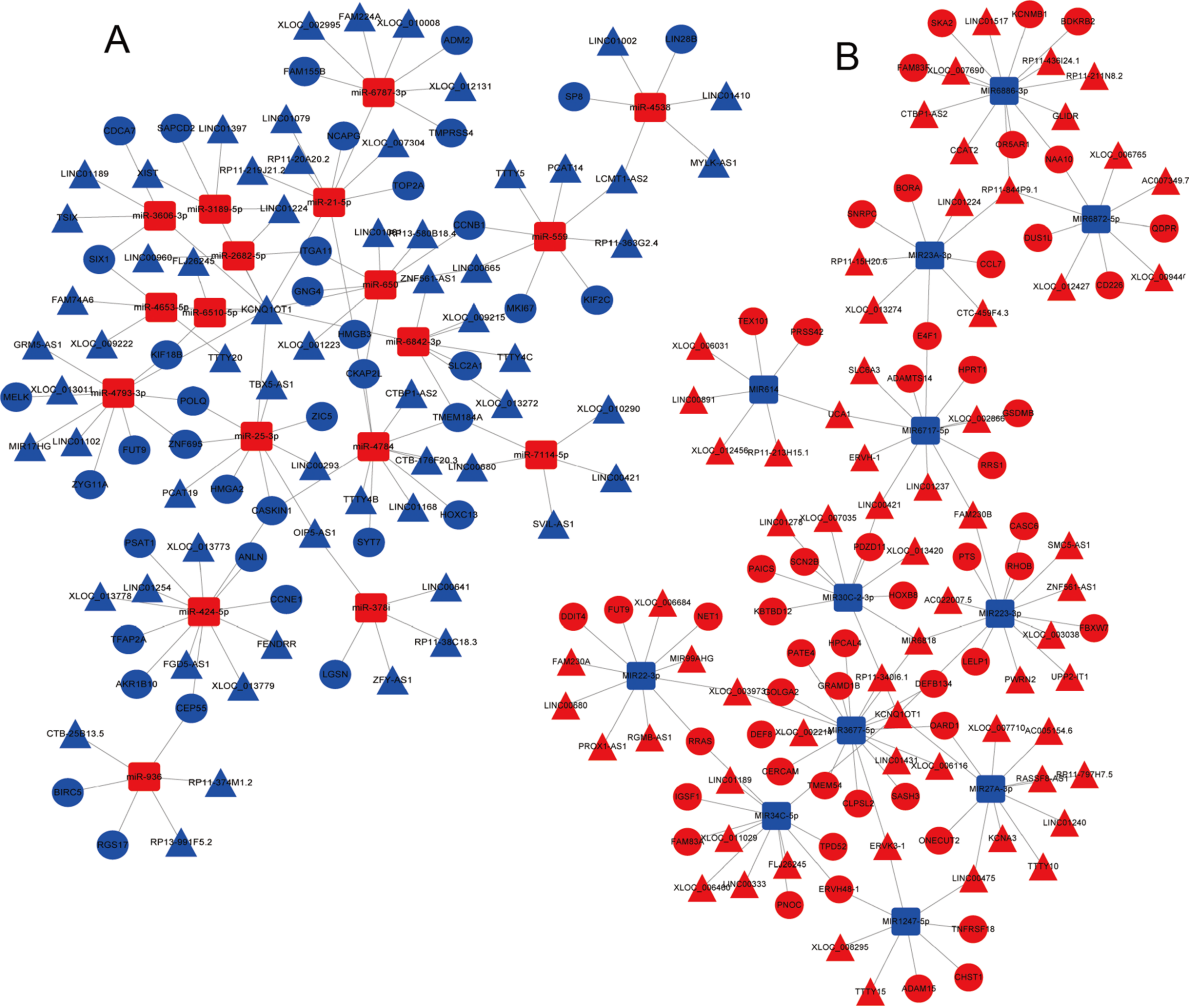
The mRNA–miRNA–lncRNA ceRNA network of over-expressed (A) and under-expressed (B) miRNAs in LUAD. Red, upregulation; blue, downregulation. Triangles represent lncRNA, circles represent mRNA, and squares represent miRNAs. miRNA, microRNA; lncRNA, long non-coding RNA; ceRNA, competitive endogenous RNA; LUAD, lung adenocarcinoma.

A total of 679 differentially expressed lncRNAs (426 upregulated and 253 downregulated) between cancerous and non-cancerous tissues were identified (Fig. 4; detailed data shown in Supplementary Table Sheet 2). LncRNAs co-expressed above in the volcano plot were selected for the construction of the ceRNA network. Predicted miRNA–mRNA and miRNA–lncRNA target sites from the dataset were identified using the results presented in Figs. 2, 3 and 4, respectively. The LUAD ceRNA network was constructed using the mRNA–miRNA-lncRNA regulation network (Fig. 5). A total of 91 mRNAs, 30 miRNAs and 125 lncRNAs were found to be co-expressed in the ceRNA network of LUAD (Fig. 5).

### Typical ceRNAs and their clinical features

**Figure 6:**
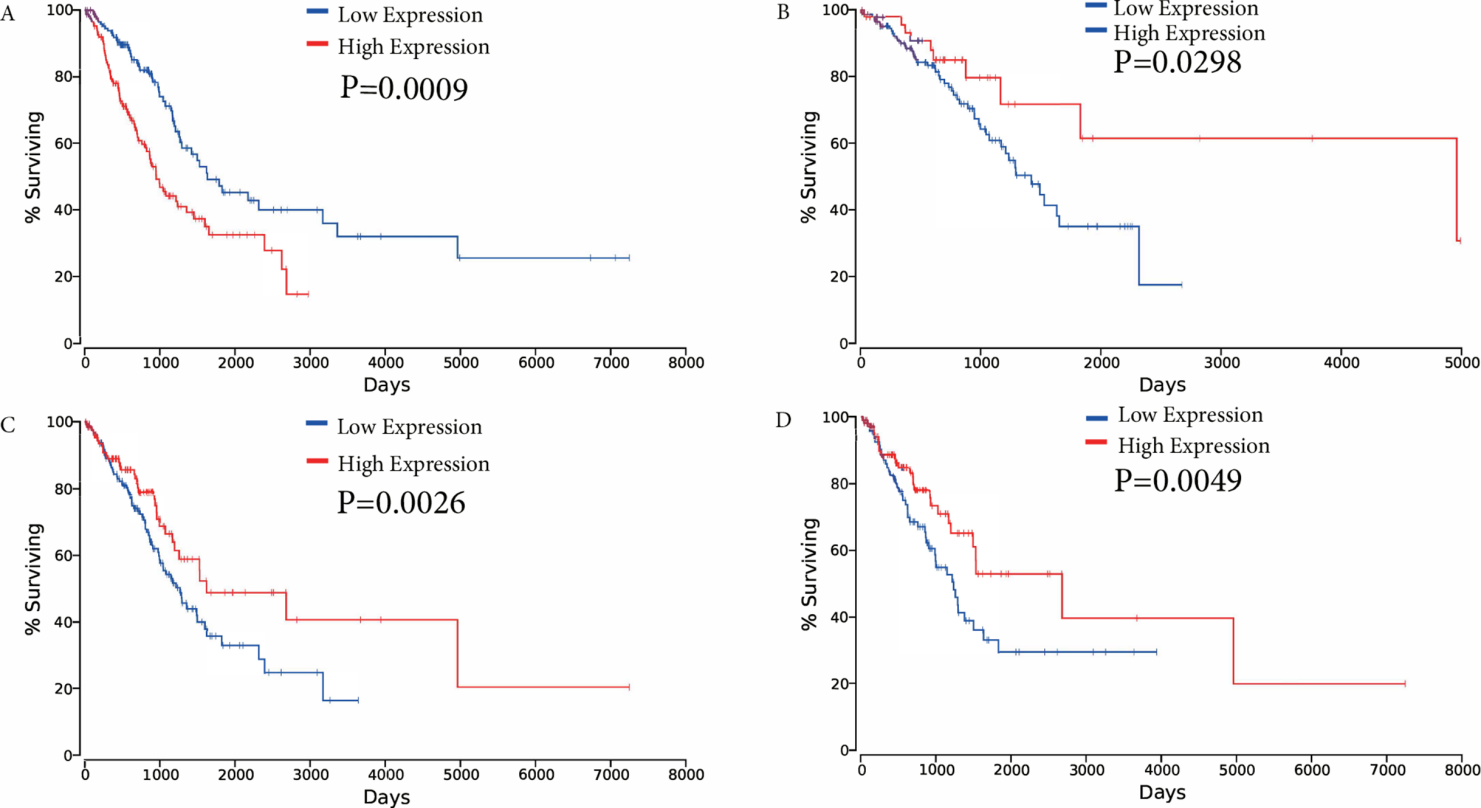
Kaplan–Meier survival curves for four ceRNAs (FAM83A, miR-34c-5p, KCNQ1OT1 and FLJ26245) significantly associated with overall survival. (A) FAM83A was negatively associated with overall survival; (B) MiR-34c-5p was positively associated with overall survival; (C) KCNQ1OT1 positively correlated with overall survival; (D) FLJ26245 was positively associated with overall survival. ceRNA, competitive endogenous RNA; FAM83A, family with sequence similarity 83 member A; miR, microRNA.

The association between overall survival and ceRNAs in LUAD patients from the TCGA database was then analyzed. Using the results of the bioinformatics analyses and the ceRNA network, ceRNAs were selected to analyze their effects on patient survival (*P* < 0.05) to determine whether specific ceRNAs are significantly associated with prognostic features. Among the differentially expressed ceRNAs, one mRNA (FAM83A), one miRNA (miR-34c-5p), and two lncRNAs (KCNQ1OT1, and FLJ26245) were found to be significantly associated with the overall survival of patients with LUAD by univariate Cox regression analysis (verified ceRNA datadata shown in Supplementary Table Sheet 3). Kaplan–Meier survival curves demonstrated that FAM83A was negatively associated with overall survival, whereas the lncRNA KCNQ1OT1, miR-34c-5p and FLJ26245 were observed to be positively correlated with overall survival (Fig. 6).

### Expression of FAM83A in LUAD

**Figure 7:**
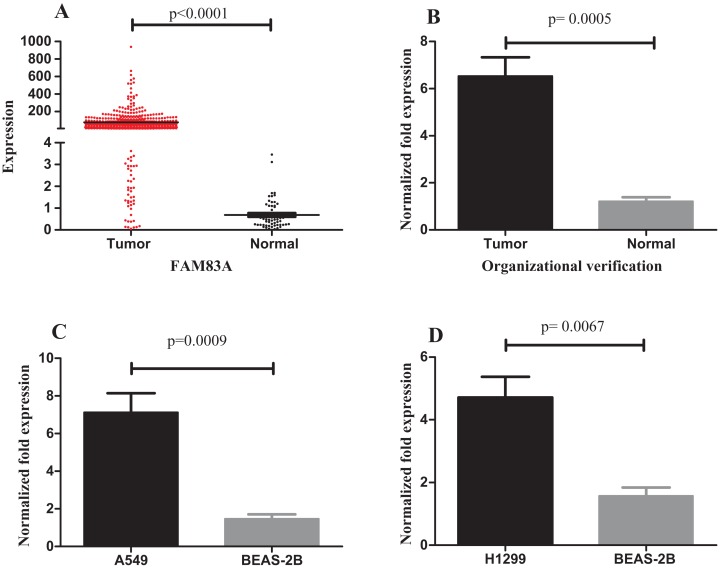
Expression of FAM83A in LUAD. (A) FAM83A was significantly upregulated in tumor groups of LUAD from the TCGA database; (B) FAM83A was significantly upregulated in LUAD tumors relative to the adjacent non-tumor tissues; (C) The expression of FAM83A in A549 cells compared with BEAS-2B cells; (D) The expression of FAM83A in H1299 cells compared with BEAS-2B cells. FAM83A, family with sequence similarity 83 member A; LUAD, lung adenocarcinoma.

The expression of FAM83A in LUAD tumor and normal samples from the TCGA database were then examined. The results demonstrated that FAM83A expression was significantly higher in tumor samples (Fig. 7A). In addition, the expression of FAM83A in 20 tumor tissue samples and adjacent tumor tissues (Fig. 7B) demonstrated that there was an eightfold difference in expression between these groups. Consistent with these observations, FAM83A transcript levels were significantly upregulated in A549 and H1299 cells when compared with BEAS-2B cells (Figs. 7C and 7D).

## Discussion

The underlying causes and successful treatment strategies for lung cancer are the focus of ongoing research worldwide (Feng et al., 2018). With the development of high-throughput sequencing technology and bioinformatics tools, the occurrence and characteristics of LUAD are gradually being elucidated. TCGA is a public database that contains a comprehensive collection of data regarding the expression of oncogenes in cancer (Tomczak, Czerwinska & Wiznerowicz, 2015). The present study reveals the latest genomic information about LUAD and has performed comprehensive multidimensional analyses using a large number of genomic sequences and TCGA datasets. The results of the current study demonstrate that LUAD is associated with tumor-specific genetic changes, characterized by alterations in gene expression profiles.

Previous studies have provided evidence to suggest that miRNAs can be considered as oncogenes or tumor suppressor genes involved in tumor development, progression, invasion and metastasis (Binmadi et al., 2018). The present study identified 107 differentially expressed miRNAs between LUAD tumor tissues and adjacent non-tumor tissues; some of which have been reported to serve a role in tumorigenesis or metastasis. Recent studies have also highlighted the role of lncRNAs in tumorigenesis, and it is thought that these lncRNAs may present suitable cancer biomarkers (Liang et al., 2018a; Ni et al., 2018). Of the 679 lncRNAs identified in this study, GAS5 levels were observed to be significantly associated with advanced clinical stage and lymph node metastasis in colorectal cancer (Cheng et al., 2018). The SNHG6 lncRNA is known to promote glioma tumorigenesis by sponging miR-101-3p (Meng et al., 2018). In addition, the ZFAS1 lncRNA has been shown to promote nasopharyngeal carcinoma progression through activation of the Wnt/β-catenin signaling pathway (Chen et al., 2018).

Previous studies have demonstrated that ceRNAs serve significant regulatory roles in the communication between different RNA transcripts and may be involved in the initiation and progression of tumors. The long non-coding RNA–HLA complex P5 (HCP5) has been found to be overexpressed in follicular thyroid carcinoma and functions as a sponge for miR-22-3p, miR-186-5p and miR-216a-5p, which activates ST6GAL2 to promote disease progression (Liang et al., 2018b). Therefore, mRNAs, miRNAs and lncRNAs may be involved in the progression and metastasis of LUAD. In the present study, the aberrant expression of mRNAs, miRNAs and lncRNAs in LUAD was analyzed using bioinformatics prediction tools and correlation analysis to generate a ceRNA network. In addition, four ceRNAs were observed to be significantly associated with the clinical features of patients; some of which have also been previously reported in the literature. For instance, FAM83A has been observed to be significantly overexpressed and associated with worse overall and disease-free survival in pancreatic cancer (Chen et al., 2017). FAM83A has been associated with metastasis and recurrence in lung cancer (Li et al., 2005). In addition, the abnormal expression of mir-34c-5p may be a promising marker for evaluating the risk of disease recurrences in patients with laryngeal squamous cell carcinoma (Re et al., 2015). Furthermore, the KCNQ1OT1 lncRNA is highly expressed in tongue squamous cell carcinoma and is associated with poor prognosis (Zhang et al., 2018a).

In the current study, upregulated FAM83A was found to be negatively associated with the overall survival of patients with LUAD, as demonstrated by Kaplan–Meier survival curve analysis. The FAM83A gene is involved in chemoresistance (Bartel & Jackson, 2017; Grant, 2012). Drug resistance has an adverse effect on the prognosis of patients; however, the mechanisms underlying the development of resistance are still unclear. The anomalous expression of FAM83A is reportedly involved in the development of drug resistance in breast cancer and cancer stem cells (Bartel & Jackson, 2017; Lee et al., 2012). However, only a small number of scholars have reported investigating the role of FAM83A in LUAD, and previous researches did not report the relevant mechanism (Li et al., 2005; Liu et al., 2008). In the present study, FAM83A was observed to be upregulated in LUAD samples when compared with adjacent non-tumor tissues. In addition, upregulated FAM83A was inversely associated with poor survival of LUAD patients, suggesting that FAM83A may present a novel therapeutic target for drug resistance in LUAD. In our present study, several limitations were identified; for example, this ceRNAs should be further verified in a large number of clinical samples and other methods such as QT-PCR or western blot.

## Conclusions

In this study, we first constructed a LUAD-associated ceRNA network consisting of 125 DElncRNAs, 30 DEmiRNAs and 91 DEmRNAs. Based on the total survival analysis, we obtained a total of four prognostic biomarkers associated with LUAD, including FAM83A, hsa_mir-34c-5p, KCNQ1OT1 and FLJ26245. We obtained a KCNQ1OT1, FJL26245–hsa-mir-34c-5p–FAM83A axis that correlates with the treatment and prognosis of LUAD through comparative integration. This further demonstrates the contribution of lncRNA, miRNA and mRNA interactions to the pathogenesis of LUAD and provides new diagnostic and prognostic biomarkers. However, further investigation is needed on the molecular pathogenesis of LUAD in order to guide the treatment and in-depth study of LUAD.

## Supplemental Information

10.7717/peerj.6809/supp-1 Supplemental Information 1 Key (GO) terms of differentially expressed intersection mRNAs. The GO enrichment plots show the enrichment counts of the significantly enriched GO terms. (A) Down-regulated GO analysis for differentially expressed genes; (B) Up-regulated GO analysis for differentially expressed genes. GO, gene ontology.

10.7717/peerj.6809/supp-2 Supplemental Information 2 Pathway enrichment analysis between tumor and normal groups of LUAD. The bar charts show the enrichment scores of the significantly enriched pathways. (A) The 20 pathways for down-regulated differentially expressed genes; (B) The 20 pathways for up-regulated differentially expressed genes. LUAD, lung adenocarcinoma.

10.7717/peerj.6809/supp-3 Supplemental Information 3 Supplemental Table.

## Additional Information and Declarations

### Data Availability

The following information was supplied regarding data availability: The raw measurements are available in the Supplemental Table.
