# Supplementary figures and images for: Identification of a putative competitive endogenous RNA network for lung adenocarcinoma using TCGA datasets
Source: PeerJ. 2019 Apr 23;7:e6809. doi: 10.7717/peerj.6809 (PMC6485208; doi:10.7717/peerj.6809)

A

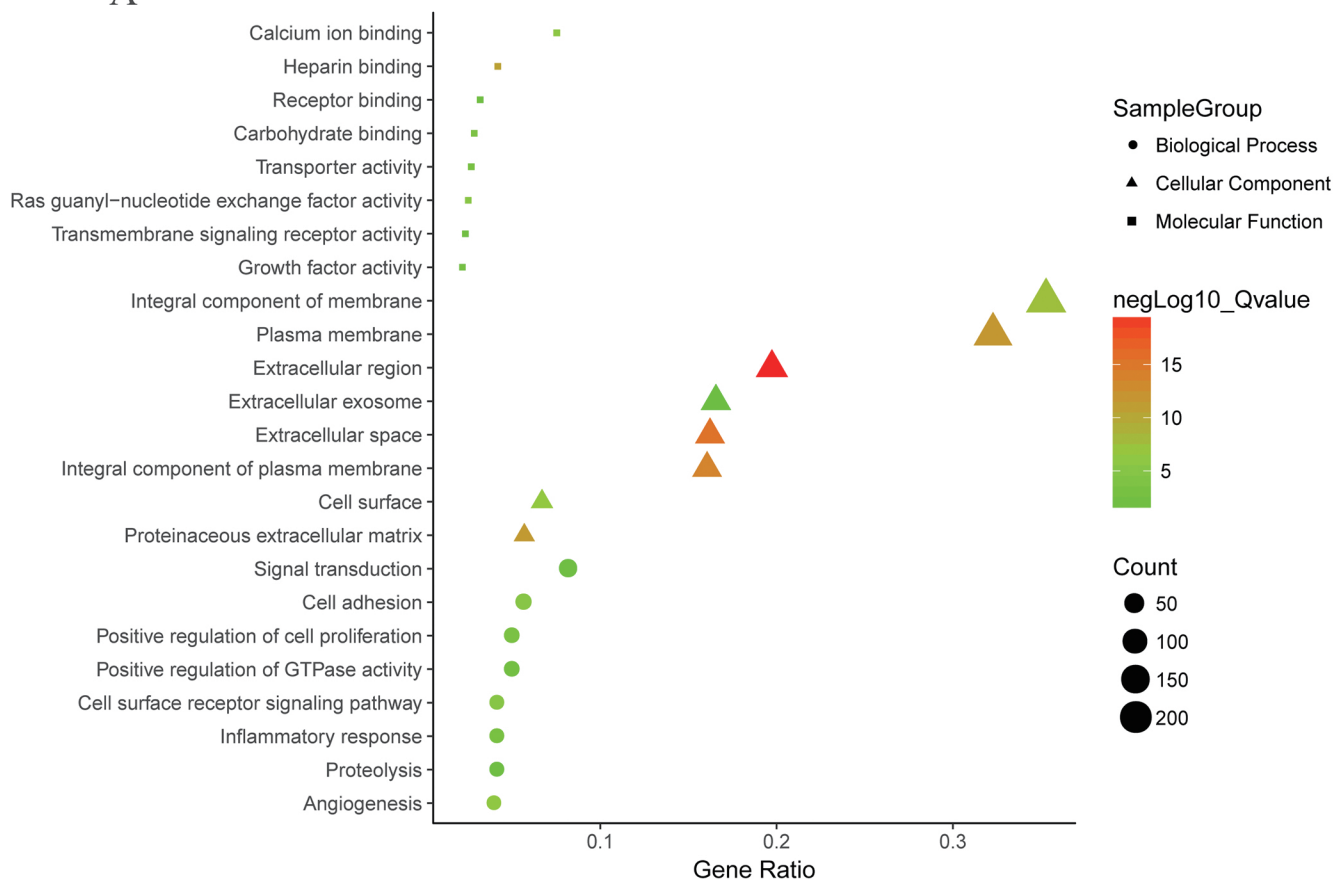

B

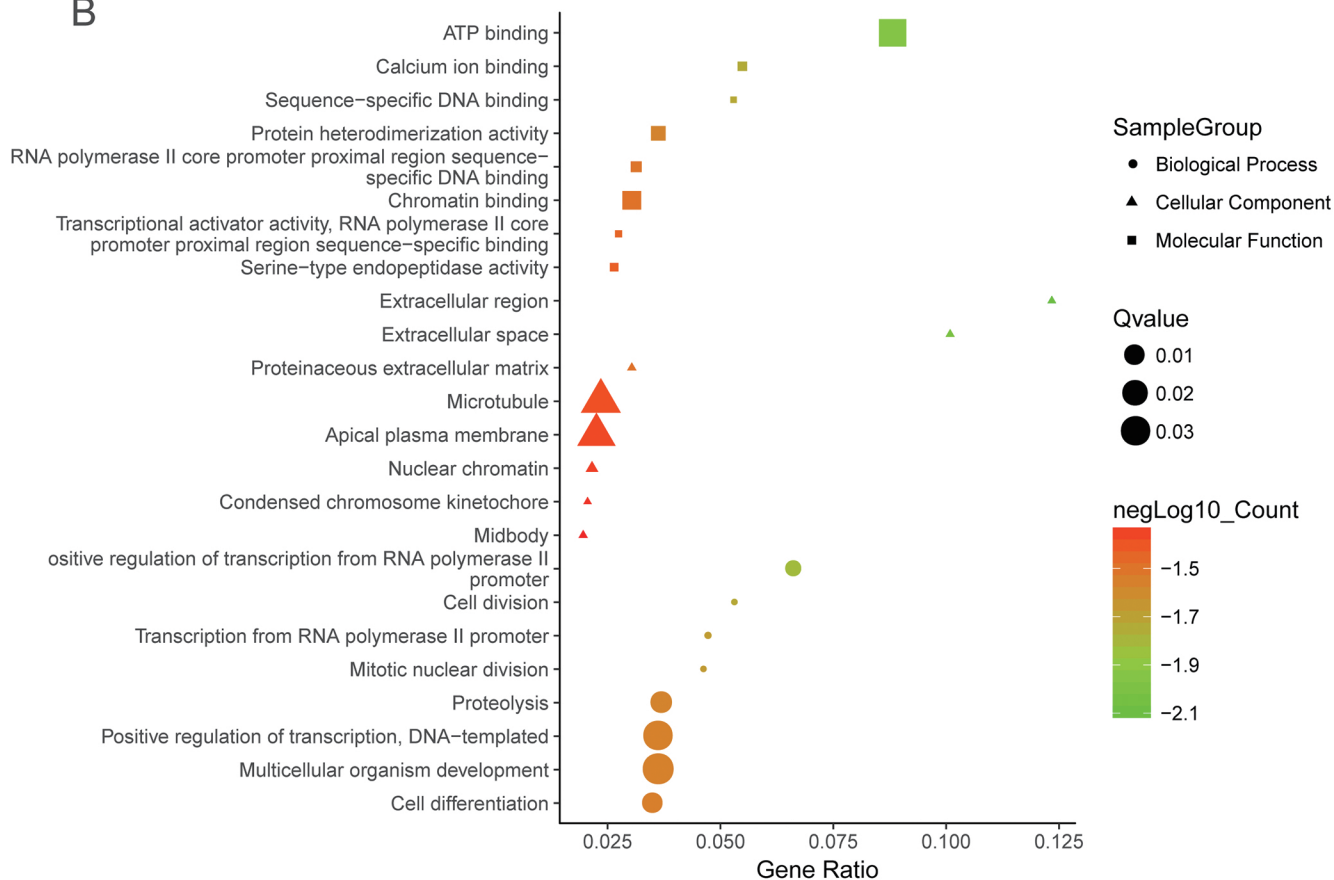

Supplement: Supplemental Information 1 — The GO enrichment plots show the enrichment counts of the significantly enriched GO terms. (A) Down-regulated GO analysis for differentially expressed genes; (B) Up-regulated GO analysis for differentially expressed genes. GO, gene ontology. [file peerj-07-6809-s001.pdf]

**(A)**

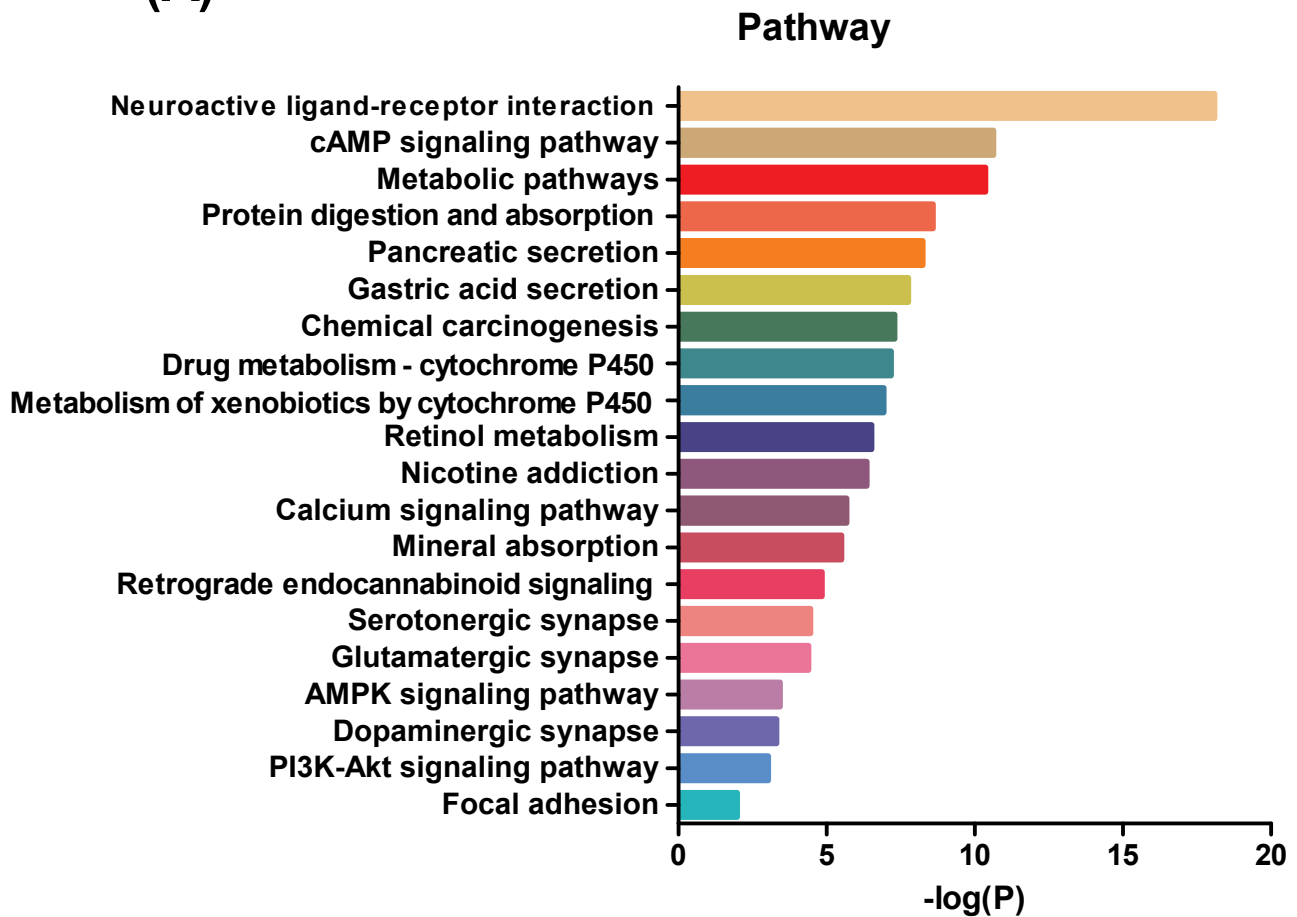

**(B)**

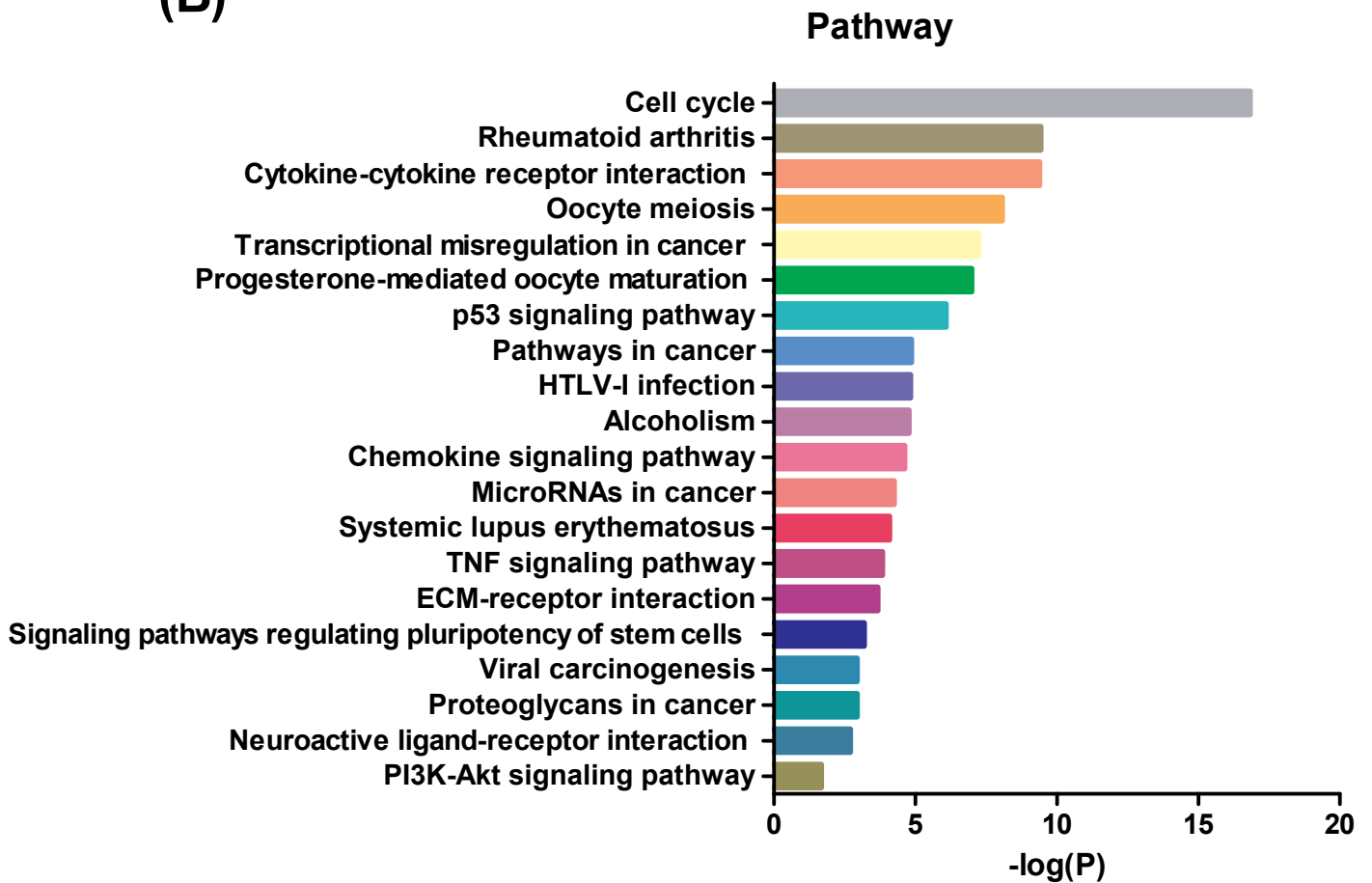

Supplement: Supplemental Information 2 — The bar charts show the enrichment scores of the significantly enriched pathways. (A) The 20 pathways for down-regulated differentially expressed genes; (B) The 20 pathways for up-regulated differentially expressed genes. LUAD, lung adenocarcinoma. [file peerj-07-6809-s002.pdf]
